# Case Report: Anti-SOX1 antibody-associated limbic encephalitis with hippocampal sclerosis: the first autopsy case

**DOI:** 10.3389/fimmu.2025.1688011

**Published:** 2025-11-27

**Authors:** Rina Izumi, Koji Hayashi, Ei Kawahara, Yasuo Miki, Koichi Wakabayashi, Asuka Suzuki, Yuka Nakaya, Naoko Takaku, Mamiko Sato, Yusuke Horiuchi, Soichi Enomoto, Yuki Kitazaki, Tadanori Hamano, Yasutaka Kobayashi

**Affiliations:** 1 Department of Rehabilitation Medicine, Fukui General Hospital, Fukui, Japan; 2 Department of Pathology, Fukui General Hospital, Fukui, Japan; 3 Department of Neuropathology, Hirosaki University Graduate School of Medicine, Hirosaki, Japan; 4 Department of Neurology, University of Fukui, Fukui, Japan; 5 Graduate School of Health Science, Fukui University of Medical Science, Fukui, Japan; 6 Department of Gerontology, Faculty of Medical Sciences, Kanazawa Medical University, Kahoku, Ishikawa, Japan

**Keywords:** anti-SOX1 antibodies, hippocampal sclerosis, Sry-like high mobility group box 1, limbic encephalitis, epilepsy, paraneoplastic, small-cell lung carcinoma

## Abstract

We report the first autopsy case of anti-SOX1 antibody-associated encephalitis accompanied by hippocampal sclerosis (HS). The patient was a 69-year-old man with a long-standing history of schizophrenia who presented with generalized seizures, fever, and altered mental status. Despite treatment for pneumonia and hyponatremia, his condition rapidly deteriorated, resulting in recurrent seizures and impaired consciousness. Imaging and cerebrospinal fluid analysis confirmed paraneoplastic limbic encephalitis associated with small-cell lung carcinoma (SCLC), and serum analysis revealed strongly positive anti-SOX1 antibodies. Following multidisciplinary interventions, palliative care was initiated, and the patient passed away on day 115. Autopsy findings included a densely proliferating SCLC in the right lung, confirming the paraneoplastic etiology. Neuropathologically, the hippocampus showed profound neuronal loss and astrogliosis primarily affecting the CA1, CA3, and dentate gyrus regions, consistent with HS. Histological analysis of the dentate gyri revealed granule cell dispersion and gliosis in the granular and molecular layers. Notably, the neuronal loss exhibited a non-classic HS pattern, distinguishing the acute pathology from typical chronic changes seen in mesial temporal lobe epilepsy or long-standing schizophrenia. CD8-positive cytotoxic T lymphocytes (CTLs) infiltrated extensively throughout the central nervous system and were particularly prominent in the hippocampus, demonstrating a T cell-mediated cytotoxic mechanism for neuronal destruction. The presence of MHC class I antigen positivity on residual neurons further supported this immune-mediated neuronal destruction. Additionally, CD8-positive CTL infiltration was observed in the sciatic nerve and mild atrophy was noted in the psoas muscle, underscoring a generalized paraneoplastic process affecting multiple tissues. These findings provide the first mechanistic pathological evidence for anti-SOX1 antibody-associated encephalitis, illustrating that neuronal destruction is driven by CD8+ CTL cytotoxicity. The case underscores the complex pathological interactions between acute autoimmune responses and pre-existing psychiatric conditions, expanding our understanding of the neuropathological spectrum of paraneoplastic limbic encephalitis associated with SCLC.

## 1 Introduction

Hippocampal sclerosis (HS) is an important neuropathological condition linked to epilepsy, which was thoroughly detailed by Sommer in 1880. He identified critical pathological alterations such as gliosis and the loss of pyramidal cells in the CA1 area of the hippocampus (1). This condition is acknowledged as a leading cause of focal epilepsy, impacting approximately 10% of adults experiencing new-onset focal seizures and frequently resulting in refractory epilepsy (2, 3).

HS can arise from multiple factors. Seizures, especially prolonged ones, make the hippocampus particularly vulnerable to injury, which can lead to neuronal loss, as observed in patients experiencing status epilepticus (4–6). Moreover, significant brain injuries occurring in early childhood, such as febrile seizures, have been associated with the emergence of HS, particularly when these injuries happen before the age of 4 to 7 years (7, 8). Genetic factors may also be significant; specific genetic variants have been linked to the incidence of both febrile seizures and HS (9). Traumatic brain injuries can also play a role in the development of HS, leading to neuronal loss and resulting seizures (10–12). Additionally, developmental issues, including minor malformations in the hippocampus, are thought to contribute to the condition (13–15). In certain instances, HS occurs alongside other cortical malformations and low-grade tumors, indicating a complex etiology (16, 17). Infections, such as viral encephalitis and neurocysticercosis, may also cause HS, especially when they impact the mesial temporal structures (18, 19). Furthermore, autoimmune disorders have been connected to this condition, with specific autoantibodies linked to changes in the hippocampus, although their exact pathogenic significance remains unclear (20–22).

Anti-SOX1 antibodies target Sry-like high mobility group box 1 (SOX1) proteins, which are crucial transcription factors involved in central nervous system development. These antibodies have been associated with a range of neurological syndromes, with Lambert-Eaton myasthenic syndrome (LEMS) being the most commonly linked condition, followed by paraneoplastic cerebellar degeneration (PCD) and sensory neuronopathy (PLE) (23, 24).

The exact mechanisms by which anti-SOX1 antibodies exert their effects are not completely understood; however, they are thought to be associated with cancers, especially small-cell lung cancer (SCLC), which is the tumor type most often found in patients with paraneoplastic neurological syndromes (25, 26). A notable number of patients with anti-SOX1 antibodies experience neurological symptoms, although some individuals may not exhibit any clinical signs even with the presence of these antibodies (27, 28). This underscores the necessity for diligent monitoring for hidden tumors, as patients with these autoantibodies may have undetected neoplasms (29).

This is the first documented case of HS proven by postmortem autopsy in a patient who succumbed to anti-SOX1 antibody-positive encephalitis associated with SCLC.

## 2 Case presentation

**Figure 1:**
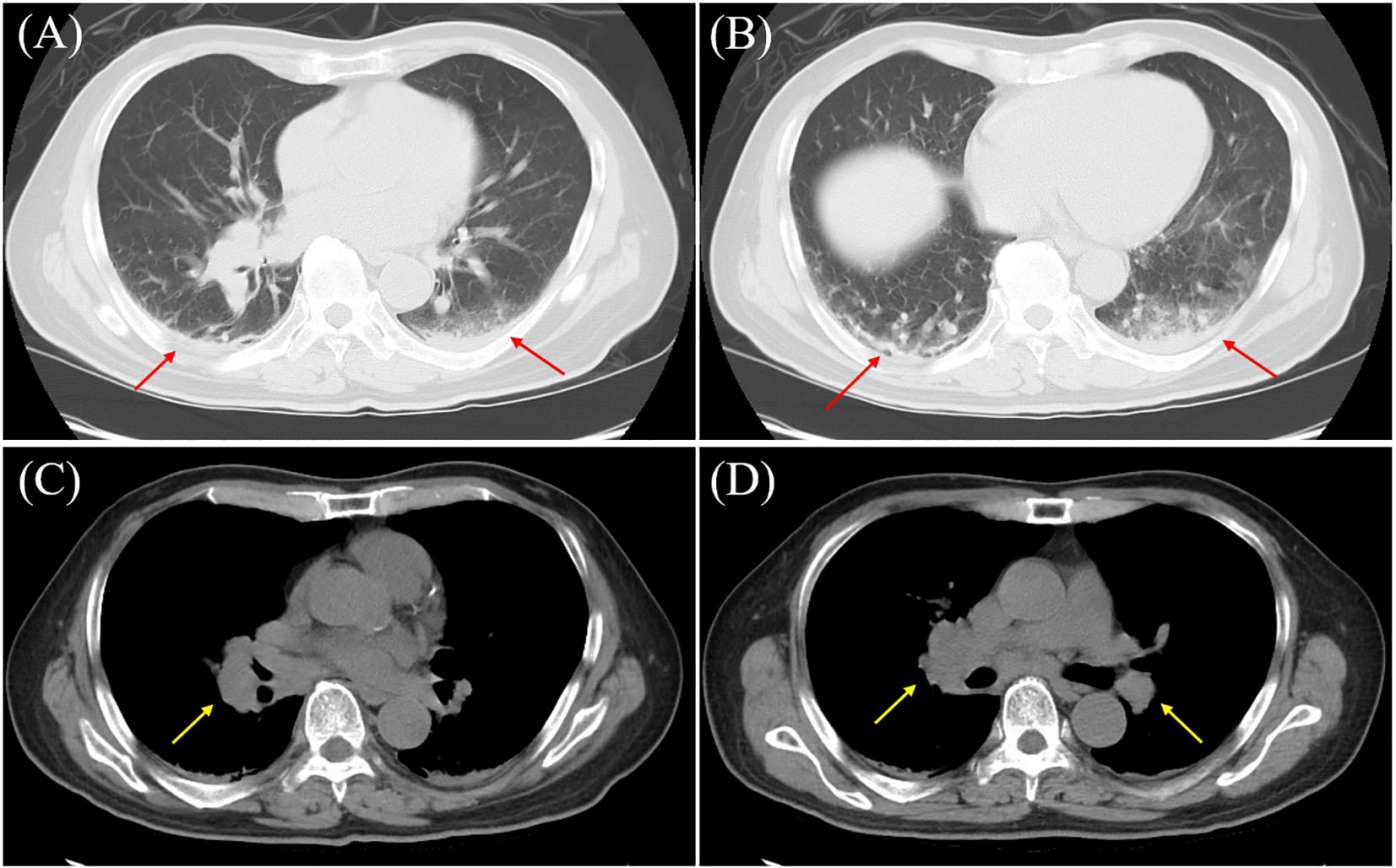
Chest computed tomography (CT) findings. Chest CT showing infiltration in the dorsal basal segment of the lower lobe (arrows), indicative of aspiration pneumonia **(A, B)**, along with mediastinal lymphadenopathy (arrows) **(C, D)**. **(A, B)** Lung window; **(C, D)** mediastinal window.

A 69-year-old man with a history of schizophrenia since age 47 (over two decades), who had been institutionalized in a psychiatric hospital, presented with generalized seizures lasting five minutes after a one-day history of fever and altered mental status. He had a history of smoking 10 cigarettes per day (though its duration was unknown) and had no prior history of epilepsy. Initial blood tests revealed a mild elevation of C-reactive protein (CRP 1.0 mg/dL), hyponatremia (Na 125 mEq/L), and a markedly elevated creatine kinase (CK 3000 U/L). Chest computed tomography (CT) showed infiltrative opacities consistent with pneumonia and hilar lymphadenopathy (**Figure 1**). Brain magnetic resonance imaging (MRI) was unremarkable on diffusion-weighted imaging (DWI).

**Figure 2:**
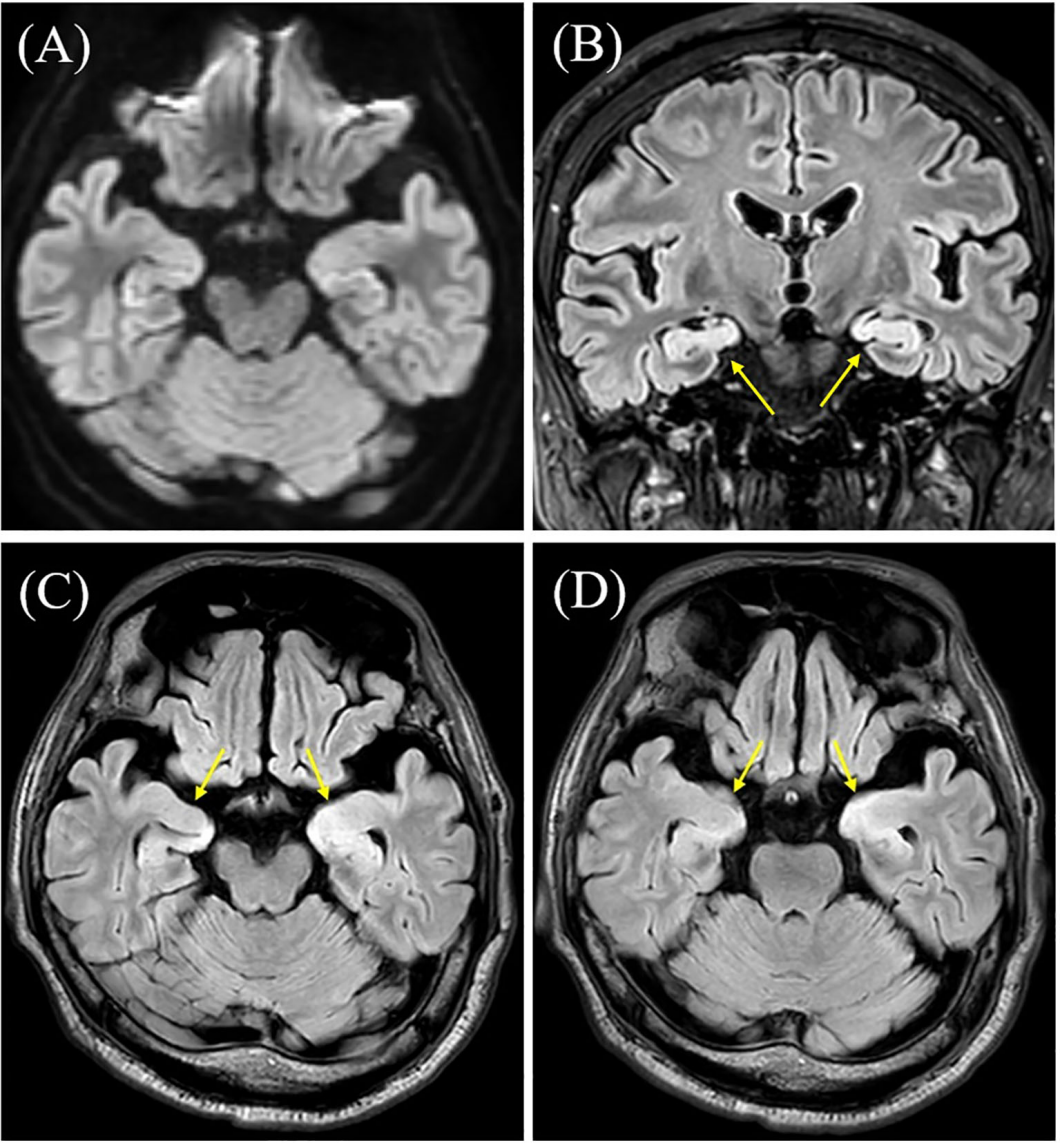
Brain magnetic resonance imaging (MRI) results. **(A)** Diffusion-weighted brain MRI was unremarkable, including the hippocampus. **(B–D)** T2 fluid-attenuated inversion recovery (FLAIR) MRI showing hyperintensity in both hippocampi (arrows). Contrast-enhanced MRI was not performed.

During the first week of hospitalization, he remained seizure-free. However, on day 8, his consciousness markedly declined, and he began experiencing recurrent, refractory seizures. A follow-up brain MRI revealed bilateral hyperintense lesions in the hippocampi on FLAIR imaging (**Figure 2**). Cerebrospinal fluid (CSF) analysis showed elevated cell counts (7/µl; reference <5/µl) and protein levels (50.2 mg/dL; reference 10–40 mg/dL), with normal glucose concentrations. On day 15, his respiratory status worsened, presenting with type 2 respiratory failure and requiring mechanical ventilation for two weeks.

**Figure 3:**
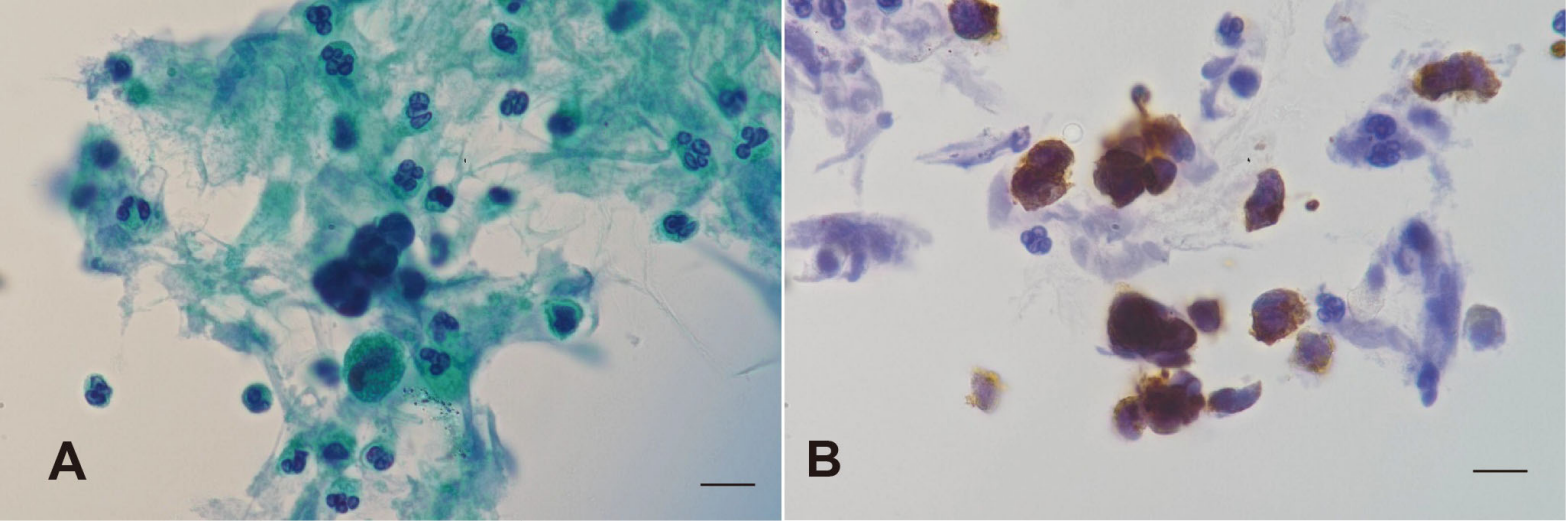
Cytopathology findings of sputum demonstrating small cell carcinoma cells. **(A)** Papanicolaou stain. **(B)** Immunoperoxidase staining for synaptophysin. Scale bar: 10 µm.

The differential diagnosis for his seizures included carcinomatous meningitis, paraneoplastic syndrome, and infectious meningoencephalitis. CSF PCR testing for HSV and VZV was negative, and cytological examination revealed no atypical cells. Serum ProGRP levels were markedly elevated (667.0 pg/mL), and sputum cytology showed atypical cells suggestive of SCLC (**Figure 3**). Analysis of paraneoplastic antibodies, including Amphiphysin, CV2, PNMA2 (Ma2/Ta), Ri, Yo, Hu, Recoverin, SOX1, Titin, Zic4, GAD65, and Tr (DNER), revealed a strongly positive anti-SOX1 antibody, assayed using the EUROLINE Paraneoplastic Neurologic Syndromes 12Ag (IgG) kit (Manufactured by EUROIMMUN [Germany]; Distributed by EUROIMMUN Japan; LOT number: D230417AN), confirming the diagnosis of paraneoplastic limbic encephalitis associated with SCLC (**Supplementary Figure S1**). The antibody was detected using immunoblotting performed by BML (Tokyo, Japan). Although a quantitative titer was not available for this method, the result was confirmed as strongly positive (++).

Given the patient’s clinical deterioration and impaired decision-making capacity, a palliative approach was chosen. Although seizures were subsequently controlled with levetiracetam or phenobarbital, the patient’s consciousness and responsiveness remained poor. He succumbed on day 115.

### 2.1 Pathological findings

**Figure 4:**
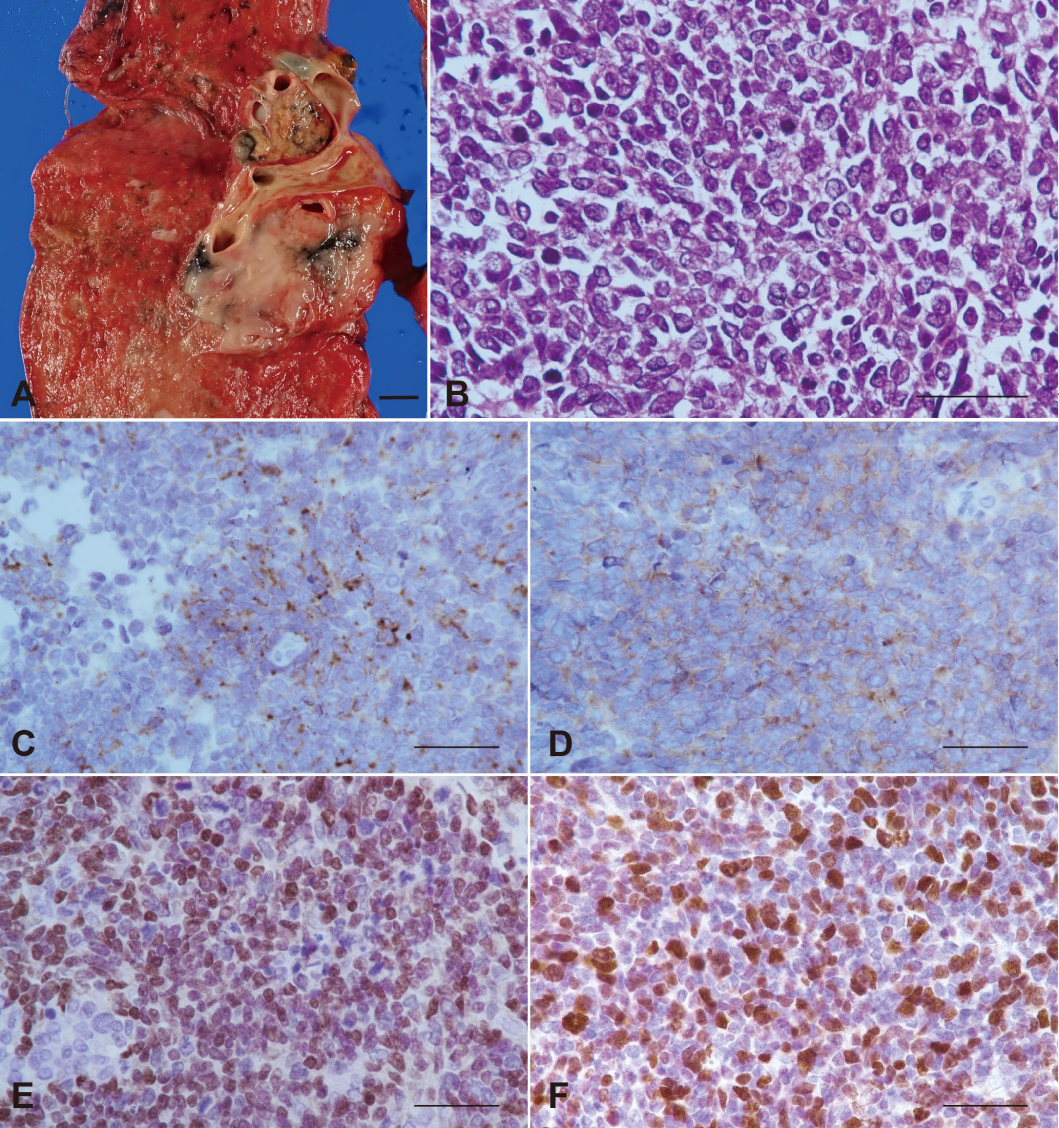
Small cell carcinoma of the lung at the autopsy. **(A)** Macroscopic feature of the tumor in the hilus of the right lung. Scale bar 1 cm. **(B)** Histopathology of the small cell carcinoma. H&E stain. Immunoperoxidase for chromogranin **(C)**, synaptophysin **(D)**, INSM1 **(E)**, Ki 67 **(F)**. **(B–F)** scale bar 50 µm.

The tumor was observed in the hilus of the right lung (**Figure 4A**). Microscopically, it was composed of solid growth of small carcinoma cells suggesting SCLC (**Figure 4B**). Immunohistochemically, the tumor cells tested positive for chromogranin A, synaptophysin, INSM1 (**Figures 4C–E**), and TTF-1. The rate of positive cell rate for Ki67 (**Figure 4F**) was 46%, all compatible with SCLC.

**Figure 5:**
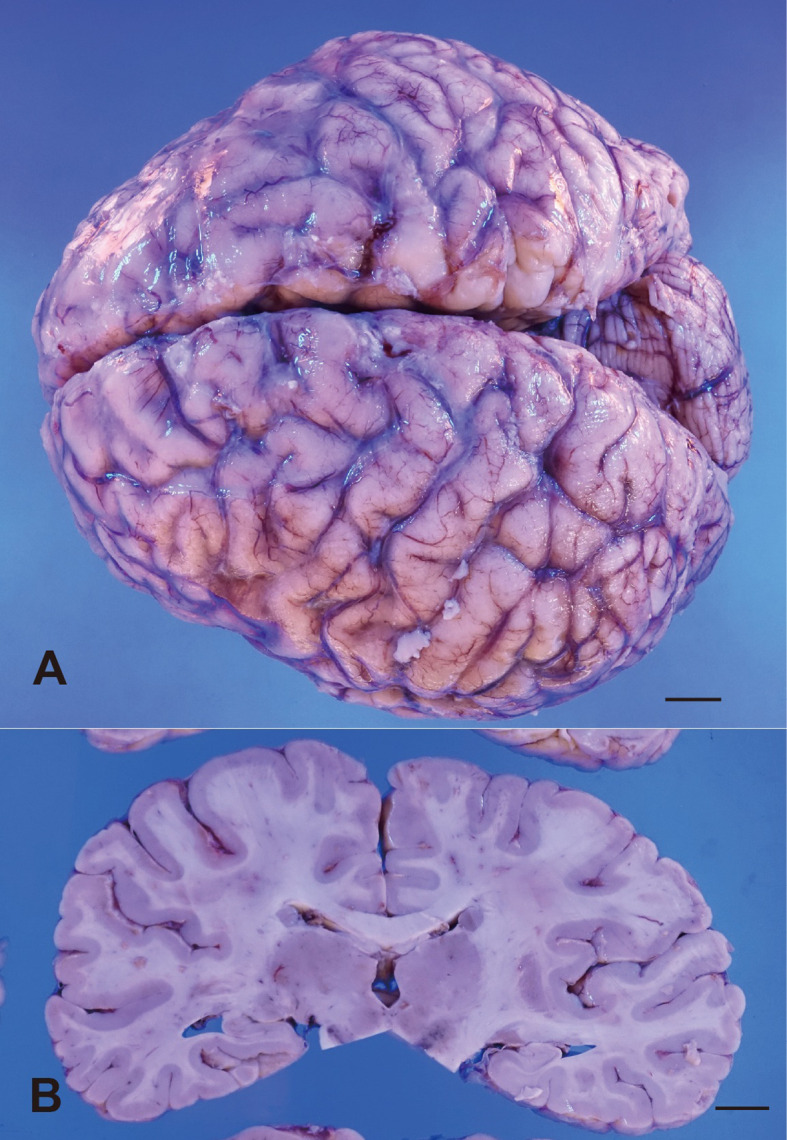
Macroscopic findings of the brain. **(A)** External appearance of the brain showing symmetrical atrophy of the cerebrum, and the arachnoid membrane is mildly cloudy. **(B)** Coronal section of the cerebrum showing mild atrophy of the hippocampus, bilateral. Scale bar, 1 cm.

**Figure 6:**
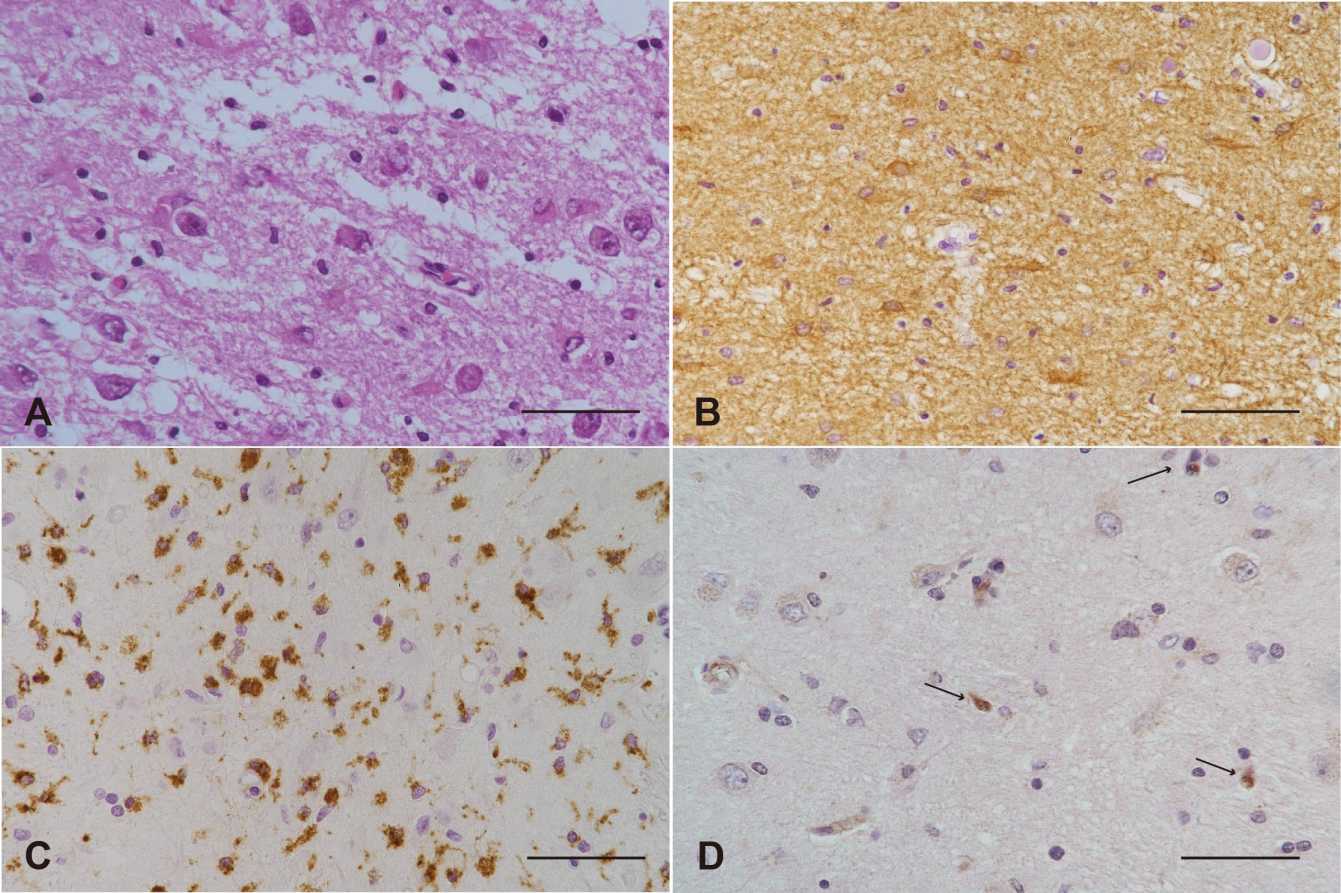
Histopathological and immunohistochemical features of the hippocampus. **(A)** Severe neuronal loss and gliosis in the CA1 region. H&E staining. **(B-D)** Immunoperoxidase staining demonstrating immune cell activation and neuronal responses. **(B)** Marked astrocytosis, indicated by strong GFAP immunoreactivity, in the CA4 region. **(C)** Increased size and number of reactive microglia, identified by CD68 immunoreactivity, in the CA1 region. **(D)** Scattered, shrunken neurons exhibiting MHC class I positivity (arrows), often attached to lymphocytes.

**Figure 7:**
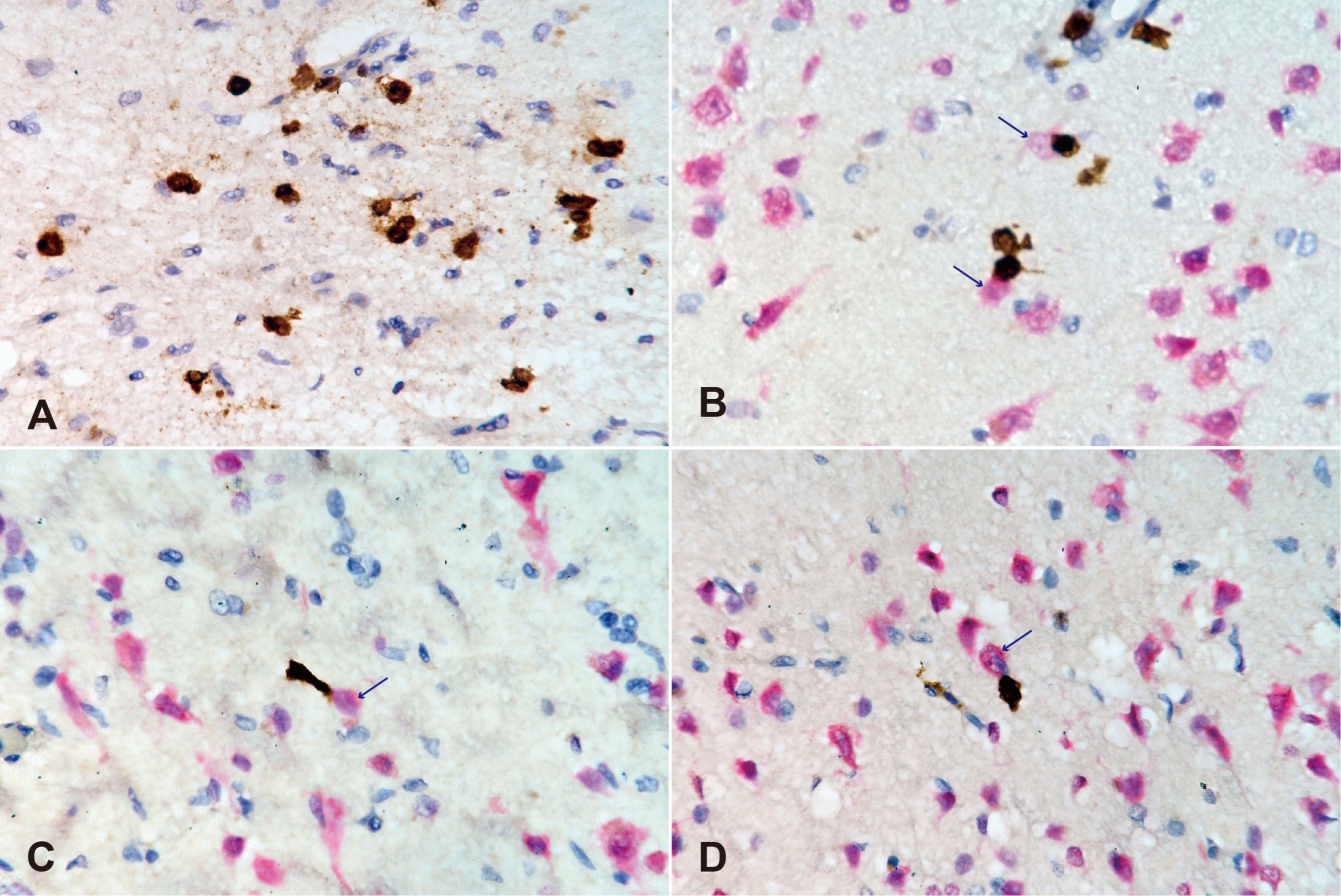
The interaction of NeuN-positive neuronal cells and CD-8 positive cytotoxic T cells. Double immunohistochemical staining using anti-mouse monoclonal CD8 antibody (brown) and anti-rabbit polyclonal NeuN antibodies (red) to visualize cytotoxic T lymphocytes and neurons, respectively. **(A)** Severe infiltration of CD8-positive cytotoxic T lymphocytes in the CA4 region, where all neuronal cells were lost. **(B)** CD8+ cells observed around a capillary (top of the image) and attached to lightly stained, shrunken neurons (blue arrows) in the CA2 region. **(C)** An elongated CD8+ cell tightly attached to a shrinking neuron (blue arrow), lightly stained for NeuN, in the CA3 region. **(D)** An amoeboid CD8+ lymphocyte attached to a shrunken neuron in the dentate gyrus. Scale bars, 50 µm.

The brain weighed 880 g and showed symmetric atrophy. The arachnoid appeared mildly cloudy (**Figure 5A**). Bilateral hippocampi were mildly atrophic on the surface (**Figure 5B**). Histologically, the hippocampus (**Figure 6**) and amygdala were the most severely affected, with severe neuronal loss on H&E (**Figure 6A**) and astrogliosis evident by GFAP (**Figure 6B**), along with increased CD68-positive microglia (**Figure 6C**), MHC class I immunostaining (**Figure 6D**), and infiltration of CD8-positive cytotoxic T cells. CD8+ CTLs were found in the parenchyma and around pericapillary areas; CD3-positive T cells localized similarly to CD8+ cells. CD20-positive B cells were detected in pericapillary regions, and MHC class I antigens were positive in a subset of neuronal cells (**Figure 6D**). Double immunostaining with NeuN and immune markers supported direct neuron–CTL interactions, with CTLs attached to neurons in elongated or ameboid forms (**Figure 7**).

**Figure 8:**
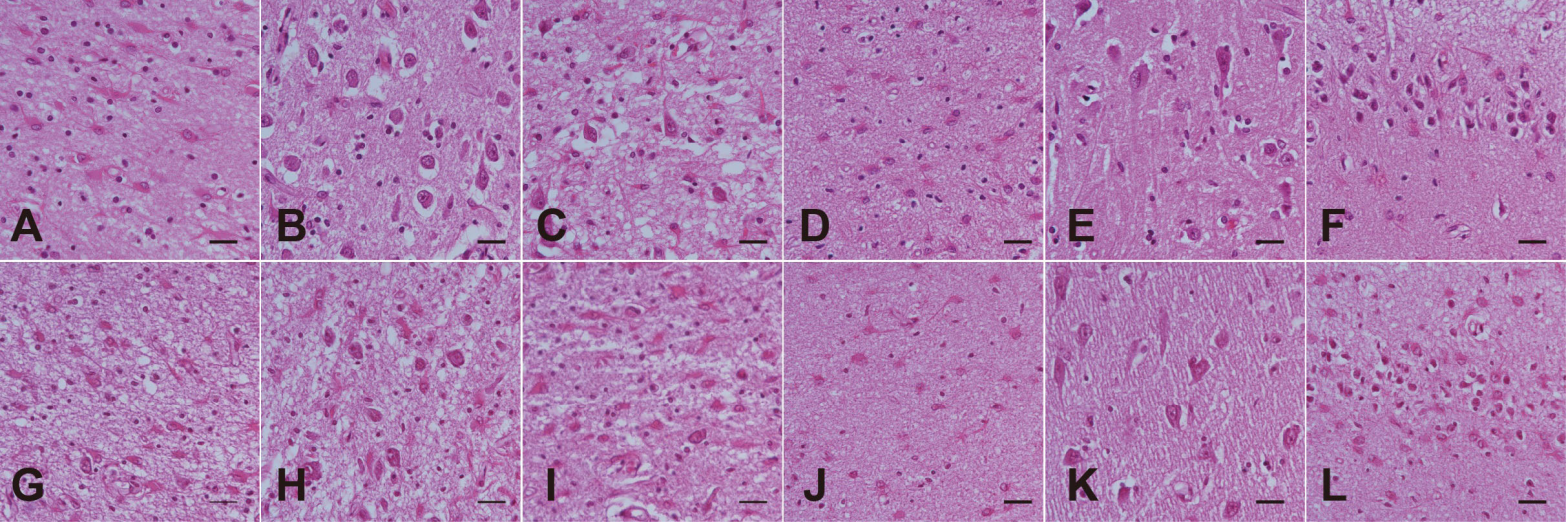
Hippocampal sclerosis in the subfields of bilateral hippocampus shown by H&E staining. **(A–F)** Left hippocampus. **(G–L)** Right hippocampus. Left CA1 **(A)**, CA4 **(D)**, and right CA4 **(K)** show complete loss of neurons and gliosis. Left CA2 **(B)**, CA3 **(C)**, right CA1 **(G)**, and right CA3 **(I)** exhibit severe neuronal loss and gliosis, with only a few neurons preserved. Right CA2 **(H)** shows moderate neuronal loss and gliosis. Both the left and right subiculum **(E, K)** show no neuronal loss. The left **(F)** and right **(L)** dentate gyrus display dispersion with marked loss of granule cells and gliosis in the granular and molecular layers. Scale bar: 50 µm.

Semiquantitative evaluation of neuronal loss was first performed using H&E staining. The results showed that the left CA1 (**Figure 8A**), CA4 (**Figure 8D**), and right CA4 (**Figure 8J**) regions exhibited complete neuronal loss and gliosis. The left CA2 (**Figure 8B**), CA3 (**Figure 8C**), right CA1 (**Figure 8G**), and right CA3 (**Figure 8I**) regions demonstrated severe neuronal loss and gliosis, with only a few neurons preserved. The right CA2 (**Figure 8H**) displayed moderate neuronal loss and gliosis. Both the left and right dentate gyri showed dispersion with marked loss of granule cells and gliosis in the granular and molecular layers (**Figures 8F**, **8L**). Neurons were well-preserved in the bilateral subiculum (**Figures 8E**, **8K**).

**Figure 9:**
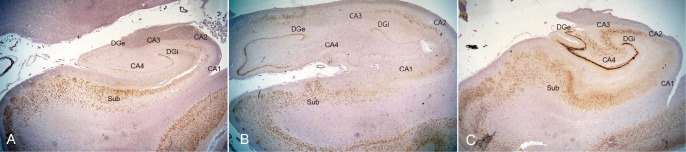
Semiquantitative presentation of neuronal loss shown by NeuN immunoperoxidase. **(A)** Anterior portion of the left hippocampus. **(B)** Posterior portion of the left hippocampus. **(C)** Less affected hippocampus of a 60-year-old individual, serving as a positive control. The right hippocampus could not be shown because immunostaining was not successful, likely due to hyperfixation. CA: Cornu Ammonis, Sub: subiculum, DGe/Dgi: the external and internal limbs of the dentate gyrus.

Immunostaining for NeuN showed that only a small portion of neurons was preserved in CA1 of the anterior portion of the left hippocampus and in CA2 of the posterior portion of the left hippocampus. NeuN immunostaining was not observed in the right hippocampus, possibly due to delayed brain sectioning of the right hippocampus, although GFAP and CD68 immunostaining were successful in the same right hippocampus. The degree of neuronal loss was evaluated only by H&E staining. The degrees of astrogliosis (GFAP) and microgliosis (CD68) paralleled the neuronal loss estimated by H&E and NeuN staining. Finally, the degrees of damage to the hippocampus, as evaluated by neuronal loss (**Figure 9**) and gliosis, according to the international consensus of hippocampal sclerosis in temporal lobe epilepsy (30), are summarized in **Table 1**.

**Table 1:** Semiquantitative microscopic examination based on hematoxylin and eosin staining, NeuN immunohistochemistry and GFAP immunohistochemistry.

Location	Lt. anterior	Lt. posterior	Rt. anterior	Rt. posterior
Subiculum	0	0	0	0
CA1	2	2	2	2
CA2	1	1	1	2
CA3	2	2	2	2
CA4	2	2	2	2
Dentate gyrus	2	2	1	1

The scoring system refers to neuronal cell loss determined by NeuN staining and H&E staining, and astrogliosis determined by GFAP staining.

CA1-CA4 and subiculum: 0 = no obvious neuronal loss or moderate astrogliosis only; 1 = moderate neuronal loss and gliosis; 2 = severe neuronal loss and fibrillary astrogliosis 2013. Dentate gyrus: 0=granule cell layer is normal; 1=dispersed; 2= severe granule cell loss, according to the international consensus classification of hippocampal sclerosis in temporal lobe epilepsy, 2013 (30).

No obvious vascular pathology, including microinfarctions, was observed in the hippocampi. Furthermore, no ischemic red neurons were observed in the hippocampus or in other brain regions, suggesting that the severe neuronal loss was not primarily attributable to acute hypoxic injury secondary to status epilepticus. Immunohistochemically, staining for phosphorylated tau, phosphorylated TDP-43, α-synuclein, and amyloid-β was negative in the brain tissue.

**Figure 10:**
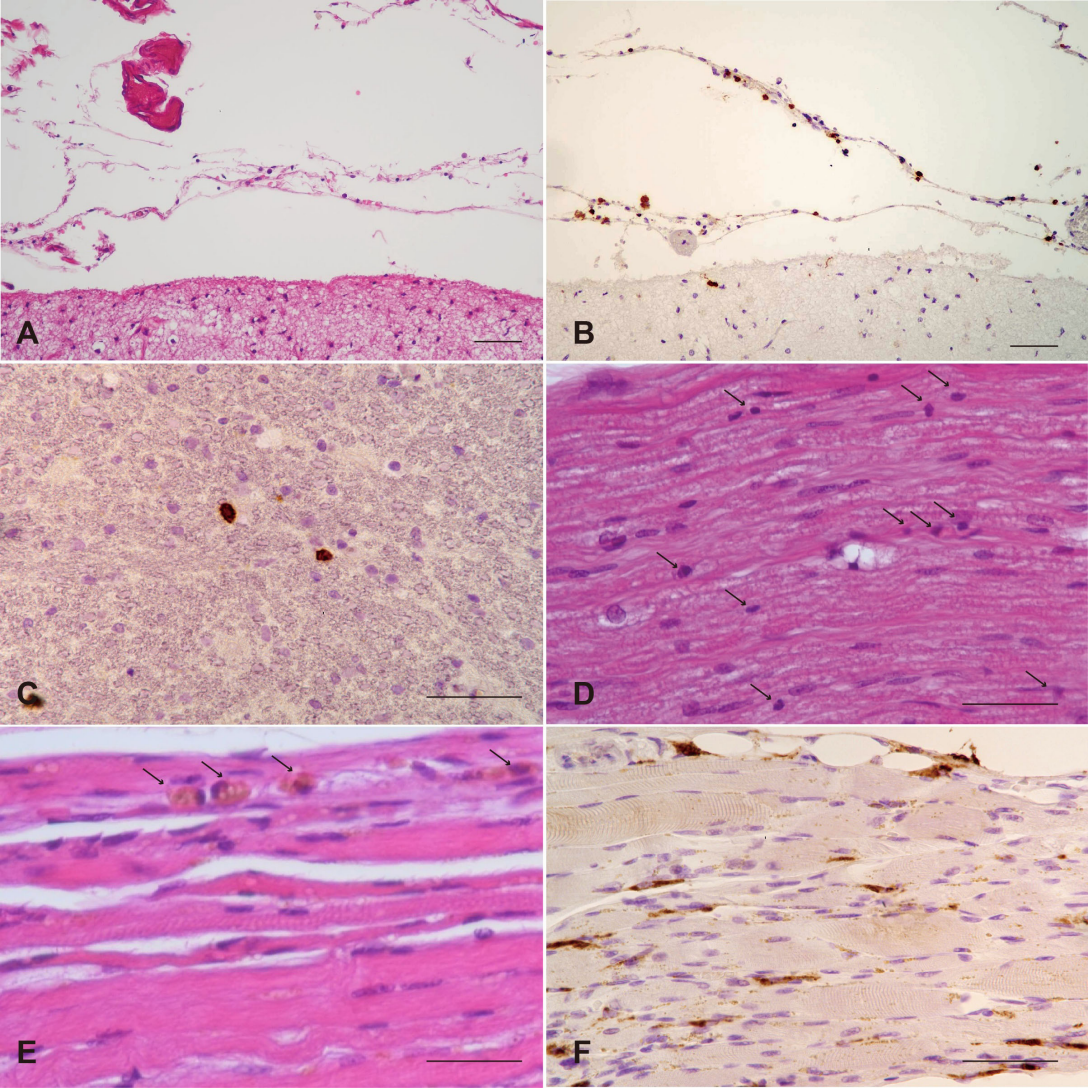
Meningeal, spinal cord, sciatic nerve, and psoas muscle pathology. **(A)** Lymphocyte infiltrates within the pia mater and arachnoid trabeculae. H&E stain. **(B)** Lymphocytes in the pia mater and arachnoid trabeculae are CD3-positive. Immunoperoxidase stain. **(C)** CD8-positive lymphocytes infiltrate the white matter of the spinal cord. Immunoperoxidase stain. **(D)** Scattered lymphocytes are present within the sciatic nerve. H&E stain. **(E)** The psoas muscle is atrophic with macrophage infiltration (arrows) and without lymphocyte infiltration. H&E stain. **(F)** CD68-positive macrophages infiltrate atrophic muscle tissue. Immunoperoxidase stain. Scale bar: 50 µm.

As listed in **Table 2**, semiquantitative microscopic examination across the whole brain revealed that severe neuronal loss (score 2) was strictly confined to the limbic system (hippocampus and amygdala). In the neocortex (frontal and lateral lobes) and the putamen, mild neuronal loss (score 1) was observed, accompanied by mild T-cell infiltration (score 1). Conversely, several regions, including the occipital lobe, subthalamic nucleus, thalamus, dentate nucleus, pons, and medulla oblongata, exhibited mild T-cell infiltration (score 1) but showed no obvious neuronal loss (score 0). No neuronal loss or T-cell infiltration (score 0/0) was evident in the globus pallidus, substantia nigra, or cerebellar hemisphere. Furthermore, lymphocytes positive for CD3 (**Figure 10B**) and CD8 infiltrated the meninges (**Figure 10A**), spinal cord (**Figure 10C**), and sciatic nerve (**Figure 10D**). In the spinal cord, they were observed in the white matter and around perivascular areas. There was evidence of interaction between spinal neuronal cell bodies and CTLs. The psoas muscles showed mild atrophy with CD68-positive macrophages (**Figures 10E, F**); however, no lymphocytic infiltration was detected in this tissue.

**Table 2:** Semiquantitative microscopic examination of the whole brain for neuronal loss and T cell infiltration.

Region	Structure	Neuronal loss	CD3+
Limbic system	Amygdala	2	2
	Hippocampus	2	2
Neocortex	Forntal lobe	1	1
	Occipital lobe	0	1
	Lateral lobe	1	1
Basal ganglia	Putamen	1	1
	Globus pallidus	0	0
	Subthalamic nucleus	0	1
	Substantia nigra	0	0
Thalamus		0	1
Cerebellar hemisphere		0	0
Dentate nucleus		0	1

The scoring system refers to neuronal cell loss determined by H & E staining, and T ell infiltration in the parenchyma by CD3 staining. 0 = no obvious neuronal loss or T cell infiltration; 1 = mild neuronal loss or T cell infiltration; 2 = severe neuronal loss or T cell infiltration.

## 3 Discussion

This report documents the first autopsy case of anti-SOX1 antibody-associated encephalitis, revealing profound pathological changes in both the central nervous system (CNS) and peripheral nervous system. We calculated the diagnostic certainty using the recently updated PNS-Care Score (31). This patient, presenting with limbic encephalitis (High-risk phenotype: 3 points), strongly positive anti-SOX1 antibodies (High-risk antibody: 3 points), and a confirmed, consistent underlying SCLC (Cancer found: 4 points), achieved a total score of 10 points. Therefore, this case meets the criteria for definite paraneoplastic neurologic syndromes (PNS). The key neuropathological finding was HS, characterized by severe neuronal loss and gliosis, concurrent with widespread CTL infiltration. This unique constellation of findings offers crucial insights into the immunopathogenesis of this paraneoplastic condition associated with SCLC.

### 3.1 Anti-SOX1 antibody and pathological mechanism

Anti-SOX1 antibodies target Sry-like high mobility group box 1 (SOX1) proteins, key transcription factors involved in neural lineage commitment and differentiation (23, 24). In adults, SOX1 expression persists mainly in neural stem cell niches, such as the subventricular zone and dentate gyrus (25, 26). These antibodies are strongly associated with SCLC and paraneoplastic syndromes, most notably Lambert–Eaton myasthenic syndrome (LEMS), cerebellar degeneration, and limbic encephalitis (27–29). Our case aligns with this, presenting with SCLC and anti-SOX1 antibody-positive limbic encephalitis.

The pathological findings overwhelmingly support a T cell-mediated cytotoxic mechanism for neuronal damage. Encephalitides caused by antibodies against intracellular antigens (such as anti-Hu) typically involve neurodegeneration mediated by CTLs (22). Since SOX1 is an intracellular antigen, its associated encephalitis is expected to involve robust T-cell cytotoxicity. We demonstrated this by showing direct neuron–CTL interactions using CD8/NeuN double immunostaining, observing CD8+ CTLs attached directly to shrunken neurons. The CTLs also exhibited shape changes—becoming elongated or amoeboid—indicating active engagement. Importantly, residual neurons expressed MHC class I antigens on their surfaces, a necessary recognition step for CTLs, providing strong evidence that neuronal loss was mediated by CD8+ CTLs (32).

### 3.2 Analysis of CNS pathology and differential diagnosis

The pathological assessment confirmed HS, characterized by severe neuronal loss, gliosis, and granule cell dispersion (GCD), predominantly affecting hippocampal subfields CA1, CA3, CA4, and the dentate gyrus (33). Given the patient’s long-standing history of schizophrenia (over two decades), this prompts an analysis of the relationship between these conditions. Schizophrenia is widely associated with hippocampal pathology, including volume reduction and interneuron loss (34, 35). The resulting astrogliosis observed in our case could reflect chronic schizophrenia-related changes. However, several findings support anti-SOX1 encephalitis as the primary, acute cause of the severe neuronal destruction:

Detailed semiquantitative scoring of the hippocampal formation (**Table 1**), which employed criteria derived from the international consensus classification of hippocampal sclerosis in temporal lobe epilepsy (30), revealed a distinct and widespread pattern of neuronal vulnerability. Specifically, severe neuronal loss (score 2) was observed consistently across the CA1, CA3, and CA4 subfields in all four quadrants examined (left anterior, left posterior, right anterior, and right posterior). Notably, the CA2 subfield also showed significant involvement, registering moderate (score 1) to severe (score 2) neuronal loss. This degree of damage in CA2 deviates from the classic mesial temporal lobe epilepsy (mTLE)-related HS pattern, where CA2 is often relatively preserved (30). Furthermore, the dentate gyrus exhibited severe granule cell loss/dispersion (score 2) in the left anterior and posterior sections, and moderate dispersion (score 1) in the right sections. Crucially, this widespread pyramidal and granule cell pathology sharply contrasted with the complete preservation of the subiculum (score 0) across all assessed sections, indicating a non-classic pattern of injury that is spatially inconsistent with typical mTLE pathology (30).

#### 3.2.1 Active inflammation

The intense and widespread infiltration of active CD8+ CTLs, coupled with the upregulated MHC class I expression on neuronal surfaces, points toward an acute or subacute T cell-mediated inflammatory process (32). The T cell infiltration was widespread across the CNS (**Table 2**).

#### 3.2.2 Exclusion of hypoxic injury

No ischemic red neurons or obvious vascular pathology were observed in the hippocampus or other brain regions, suggesting the severe neuronal loss was not primarily attributable to acute hypoxic injury secondary to the refractory seizures the patient experienced.

#### 3.2.3 HS pattern

The observed pattern of neuronal loss (severe loss across CA1, CA3, CA4, and the dentate gyrus, with preservation of the subiculum) is characteristic of a non-classic HS pattern. While chronic schizophrenia may contribute to baseline vulnerability or gliosis (34, 35), the aggregate findings support the acute anti-SOX1 antibody–associated encephalitis as the driver of the severe HS observed at autopsy, rather than HS secondary to chronic structural pathology typically seen in mTLE (33).

Autoimmune limbic encephalitis related to antibodies against intracellular antigens (Hu, Ma2, GAD) or surface antigens (VGKC) can cause HS, characterized by neuronal loss and atrophy (21, 22). Our case, therefore, likely represents a T-cell-driven autoimmune etiology superimposed on a pre-existing condition, leading to severe HS and acute clinical deterioration.

### 3.3 Widespread and peripheral involvement

The pathological process was not confined to the limbic system. Our brain-wide analysis (**Table 2**) demonstrates widespread CD3-positive T-cell infiltration across the CNS, including the putamen, thalamus, and neocortex. Crucially, we found involvement of the peripheral nervous system, with CTL infiltration observed in the sciatic nerve and meninges (**Figure 10**). This peripheral finding aligns with the known clinical associations between anti-SOX1 antibodies and peripheral syndromes, such as LEMS and sensory neuronopathy (28, 29, 36, 37). While myasthenic symptoms remain uncertain due to the patient’s long institutionalization, the presence of CTLs in the sciatic nerve strongly suggests a generalized, multifocal paraneoplastic process extending beyond the brain. Although CD20-positive B cells were detected in pericapillary regions, the central mechanism remains predominantly cytotoxic T cell-mediated.

### 3.4 Limitations

There are some limitations in this report. First, no banked serum prevented cell-based assays (CBA) for neuronal surface antibodies (e.g., anti-GABABR, anti-AMPAR1/2) and anti-SOX1 serology; P/Q-type VGCC testing to exclude subclinical LEMS/ataxia was not possible. Second, dorsal root ganglia (DRG) tissue was unavailable for pathology, hindering confirmation of CD8/NeuN cytotoxicity to sensory neurons. Third, no contrast-enhanced brain MRI to exclude SCLC brain metastases. Fourth, as our facility is a community hospital without research funding, we were unable to procure certain pathological reagents. Therefore, overall data/autopsy sampling constraints limit interpretability of peripheral and immunopathogenic aspects. Had these results been included, the presentation would have been more compelling.

## 4 Conclusion

We present the first autopsy case of a patient with anti-SOX1 antibodies, revealing significant findings in the brain, including HS and limbic encephalitis. Additionally, there was notable inflammatory cell infiltration in the peripheral nerve. These findings align with previously documented clinical symptoms associated with anti-SOX1 antibodies, underscoring the multifaceted nature of this condition. Further studies are needed to elucidate the pathogenesis of anti-SOX1 antibodies and their clinical spectrums.

## Data Availability

The original contributions presented in the study are included in the article/**Supplementary Material**. Further inquiries can be directed to the corresponding author.

## Supplementary material

Supplementary Figure 1 High-resolution scan of the stored immunoblot strip showing strong positivity for SOX1 antibodies.
