# Supplementary figures and images for: Case Report: Anti-SOX1 antibody-associated limbic encephalitis with hippocampal sclerosis: the first autopsy case
Source: Front Immunol. 2025 Nov 27;16:1688011. doi: 10.3389/fimmu.2025.1688011 (PMC12696179; doi:10.3389/fimmu.2025.1688011)

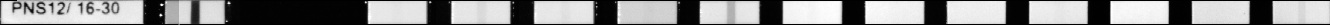

Supplement: Supplementary Figure 1 — High-resolution scan of the stored immunoblot strip showing strong positivity for SOX1 antibodies. [file Image1.jpeg]
